# Sexual Orientation and Gender Identity (SOGI): A Tutorial on Ethical Data Practices

**DOI:** 10.1007/s40617-024-01014-z

**Published:** 2025-02-04

**Authors:** Fernanda S. Oda, Christie M. Stiehl

**Affiliations:** 1 Department of Psychology, University of the Pacific, 3601 Pacific Ave., Stockton, CA 95211 USA; 2 Stiehl Behavioral Consulting, LLC, Stockton, CA USA

**Keywords:** Sexual orientation, Gender identity, Ethics, LGBTQ, DEI, Cultural humility

## Abstract

In the U.S., approximately 13 million individuals identify as part of a sexual and gender minority (SGM). This broad spectrum includes sexual orientation identities such as *gay*, *lesbian*, *bisexual*, *queer*, *pansexual*, and *same-gender-loving*, as well as gender identities such as *transgender*, *gender non-conforming*, *bigender*, and *two-spirit*. As behavior analysts heed the call to engage in culturally competent practices that address diverse sexual orientation and gender identities (SOGI), they will likely consider collecting SOGI data as part of their practice. The benefits of SOGI data collection certainly exist. However, the historical oppression and increased vulnerability of SGM populations require a careful and thorough evaluation of ethical data collection practices to avoid harm and to ensure respectful and inclusive practices. The present tutorial aims to begin the discussion of ethical and effective SOGI data collection practices within behavior analysis by offering initial guidelines and considerations. We highlight methods that improve cultural sensitivity, and caution against traditional methods that could harm respondents or contribute to a non-inclusive environment.

Collecting data about sexual orientation and gender identity (SOGI) is often undertaken in response to demands for culturally responsive approaches that prioritize inclusivity and understanding of diverse identities within communities and institutions (Wright, 2019). Behavior analysts across disciplines regularly encounter opportunities to engage with demographic data, whether they are clinicians learning about new clients, administrators surveying their staff, or researchers categorizing their participants’ data. In these contexts, behavior analysts must evaluate if and how they will include information about respondents’ sex, gender, and sexual orientation. Given that the dominant discourse about these topics is still grounded in binary measures (Rushton et al., 2019), professionals may overlook the complexity of these constructs and the ongoing cultural competence necessary to address these topics effectively (National Academies of Sciences, Engineering, and Medicine, 2022). With soundly designed procedures and a clear rationale, SOGI data collection can both validate respondents’ identities and yield rich information related to these core identities. Conversely, poorly designed SOGI data procedures risk harming sexual and gender minority (SGM) respondents and can result in the collection of misrepresentation or data that may not accurately reflect the diversity of SOGIs.

SGM individuals, also referred to as LGBTQIA2S+ (i.e., Lesbian, Gay, Bisexual, Transgender, Queer/Questioning, Intersex, Asexual, Two-Spirit, and additional identities), include those whose sexual orientation or gender identity does not align with societal expectations based on their sex assigned at birth (Capriotti & Donaldson, 2022). Over 13 million people in the U.S. self-identified with at least one SGM identity (State Health Access Data Assistance Center, 2024), although this is likely an underestimation given the considerations we discuss later in this paper. Regardless, it is well understood that the U.S. SGM population is growing in number and gaining more visibility (National Academies of Sciences, Engineering, and Medicine, 2022). Within the field of behavior analysis, comprehensive SGM data for behavior analysts and client populations do not yet exist. The Behavior Analyst Certification Board (BACB) collects data on certificants’ gender identity by offering five multiple-choice options: “male,” “female,” “nonbinary,” “prefer not to answer,” and “other.” As of January 2024, 0.63% certificants identified as nonbinary, 0.07% as “other,” and 1.00% preferred not to answer (BACB, n.d.). In addition, the primary population served by ABA practitioners, autistic individuals, are more likely to report an SGM identity compared to the neurotypical population (Dattaro, 2020; Warrier et al., 2020). As such, most behavior analysts have and will continue to engage with SGM individuals as part of their professional practice.

Recognizing the critical need for culturally sensitive practices, behavior analysts have begun calling for discussions to improve cultural competence and cultural humility in the field (e.g., Arango & Lustig, 2022; Beaulieu et al., 2019; Beaulieu & Jimenez-Gomez, 2022; Fong et al., 2016; Fong & Tanaka, 2013; Gatzunis et al., 2022; Jimenez-Gomez & Beaulieu, 2022; Li et al., 2023; Pritchett et al., 2021, Wright, 2019). Similarly, the BACB mandated that behavior analysts address diversity with compassion, dignity, and respect (e.g., BACB, 2020; Code 4.07, Core Principle 2). However, few resources exist to support this initiative aside from some discussion articles (e.g., Capriotti & Donaldson, 2022; Conine et al., 2022; Johnson, 2022; Martinez & Mahoney, 2022; Morris et al., 2021) and a few applied resources (e.g., Leland & Stockwell, 2019; Petronelli & Ferguson, 2022). To date, the authors are not aware of any behavior-analytic articles focused on ethical SOGI data practices. The current paper aims to begin the discussion of ethical and effective SOGI data collection practices within behavior analysis by offering initial guidelines and considerations.

A note to readers: As with any recommended practices, these guidelines must be evaluated within the reader’s specific applied context and decisions must be made using well-informed and sound judgment. It is important to note that this tutorial includes recommendations that the authors believe are appropriate for the current cultural context and their own lived experiences professionally and personally. These recommended practices, along with the verbal behavior used to describe them, will inevitably become outdated as verbal communities change. Readers must remain cognizant of these changes and adjust the guidelines to fit their own contexts and verbal communities. In addition, given that recommended practices are influenced by one’s experiences and values, the authors wish to acknowledge that their own identities influenced the conception, content, and structure of this paper (Parson, 2019). Fernanda’s (she/her) identities include woman, queer, Brazilian, Japanese, Latina, Asian, able-bodied, international scholar in the U.S., and native Portuguese speaker. Through her personal and professional journey, she has experienced marginalization and discrimination. Consequently, she acknowledges that her research interests are motivated by her history of learning. *Christie (they/them)* identifies as nonbinary, queer, transmasculine, white, able-bodied, U.S. citizen, and native English speaker. Although both authors hold identities classified as SGM and promote social justice within their work, they cannot fully understand or represent the experience of all members of these minority groups. The following content is based on a review of the current literature as well as their own perspectives and lived experiences pertaining to SGM topics.

## Defining SOGI Terms: A Behavior-Analytic Conceptualization

The first step toward ethically collecting SOGI data is to establish familiarity with the common terminology of SGM populations. A typical error of survey designers who have not established this familiarity involves conflating the terms *sex* and *gender* instead of recognizing them as distinct concepts (Suen et al., 2020). As verbal behavior about SOGI evolves rapidly, maintaining familiarity with terminology requires regular and ongoing contact with resources documenting cultural changes (American Psychological Association [APA], 2020). In addition to terminology changing over time, specific terms may have multiple definitions or connotations across verbal communities. For example, the term *queer,* in reference to SGM individuals, began as a pejorative term in the 1970s. Since then, younger generations have reclaimed the term and used it to empower those expressing a broad range of gender identities, gender expressions, and sexual orientations (Coles, 2016). However, this term continues to offend some SGM individuals, particularly those who first experienced this term as hate speech (Rocheleau, 2019). Conversely, previously accepted terms, such as *homosexual* and *transexual*, have fallen out of favor in most SGM communities (APA, 2020).

Table 1 lists commonly accepted definitions of the concepts *sex*, *gender identity*, *gender expression*, and *sexual orientation*, and examples of identities within each domain. *Sex* refers to biological sex assigned at birth (APA, 2011; Short et al., 2013). Medical professionals assign an infant’s sex as male, female, or intersex typically based on genitalia appearance (Raveenthiran, 2017), although there are other biological indicators (e.g., sex chromosomes, gonads, external genitalia, internal reproductive organs) that can account for the biological complexity of sex (APA, 2011). In contrast, *gender* does not rely on biological indicators but instead on social constructs (Short et al., 2013). Gender refers to attitudes and feelings assigned to people of a specific sex by the dominant culture (APA, 2015, 2020). Expanding further, gender branches out into additional concepts, including gender identity and gender expression. *Gender identity* refers to a person’s internal sense of their gender (APA, 2020) while *gender expression* refers to the way a person communicates their gender through behavior, clothing, and appearance (APA, 2011; Beltz et al., 2021). *Sexual orientation* refers to an individual’s enduring physical, romantic, or emotional attraction to another person (APA, 2020; Roselli, 2018). *Cisgender* describes individuals whose gender identity aligns with their sex assigned at birth (APA, 2015). *Heterosexual* and *straight* are terms commonly used to refer to cisgender men exclusively attracted to cisgender women and vice versa (APA, 2015). *Queer* and *transgender* are umbrella terms. *Queer* broadly describes individuals who hold one or more SGM identities. *Transgender* is associated with gender identities or gender expressions that do not conform to the individual’s sex (APA, 2015). As dynamic concepts, SOGI identities are not exclusive or static. An individual may not classify their SOGI with any identity labels, they might identify with multiple identities, or they may adjust their identities based on context or across their lifespan.

**Table 1:** Sexual orientation and gender identity: concepts, definitions, and examples

Concept	Definition	Behavior-analytic interpretation	Examples
***Sex***	Biological sex assigned at birth. Medical professionals assign an infant’s sex based on biological indicators, such as sex chromosomes, gonads, external genitalia, and internal reproductive organs (APA, 2011; Short et al., 2013)	Tacts, emitted by medical professionals, influenced by nonverbal stimuli (e.g., external genitalia) of the described individual	*Female*, *intersex*, *male*
***Gender identity***	A person’s internal sense of their gender (APA, 2011, 2020)	*Verbal* behavior (overt or covert) *about own* behaviors associated with their own gender	*Girl, woman, female, cisgender female, boy, man, male, cisgender male, agender, nonbinary, transgender, queer, transgender and gender-nonconforming (TGNC), genderqueer, gender-nonconforming, gender-neutral, gender-fluid, two-spirit, bigender, pangender, neutrois, demigender*
***Gender expression***	The way a person communicates their gender through behavior, clothing, and appearance (APA, 2011; Beltz et al., 2021)	Someone’s *nonverbal* or *overt verbal* behaviors about gender identity maintained by social contingencies and expressed to others	*Queer, Transgender, TGNC, masculine, feminine, nonbinary, transmasculine, transfeminine*
***Sexual orientation***	An individual’s enduring physical, romantic, or emotional attraction to another person (APA, 2011, 2020; Roselli, 2018)	Someone’s verbal behavior about their *own* feelings, thoughts, and attraction *toward others,* primarily controlled by own biological and physiological variables	*Asexual*, *bisexual*, *gay*, *heterosexual*, *lesbian*, *multisexual*, *omnisexual*, *pansexual*, *polysexual*, *queer*, *straight*, *same-gender-loving*, and *questioning*

This conceptualization describes verbal behavior for interpretative purposes only. Attempting to manipulate an individual’s gender identity, gender expression, or sexual orientation (i.e., “conversion” and “reparative” therapies) causes significant harm and is widely understood to be unethical

Table 1 offers a behavior-analytic interpretation of these terms through an operant framework based on Skinner’s (1957) conceptualization of verbal behavior. Please note that this analysis describes behaviors for interpretative purposes only. Attempting to manipulate an individual’s gender identity, gender expression, or sexual orientation (i.e., “conversion” and “reparative” therapies) causes significant harm and is widely understood to be unethical (e.g., APA, 2021; Association for Professional Behavior Analysts [APBA], 2024). As a foundational principle in the BACB Ethics Code, behavior analysts must treat others equitably and must respect self-descriptive verbal behavior that describes identities. *Sex* can be described as a tact emitted by medical professionals that is influenced by nonverbal stimuli (e.g., external genitalia) of the described individual. In contrast, gender identity and expression refer to covert or overt verbal behaviors emitted by the described individual. *Gender identity* can be defined as *verbal* behavior, covert or overt, by the described individual *about their own* behaviors associated with their own gender. As with any verbal behavior, responses related to SOGI can serve different functions and be controlled by multiple variables depending on the context in which they are emitted (Michael et al., 2011). It is possible to interpret that private events related to biological and physiological variables are controlling stimuli of verbal behavior about gender identity. Skinner (1974) described that there is a small part of the universe within the skin of each of us. These internal stimuli, which can include biological variables related to sex, can acquire stimulus function (Tourinho, 2006). Individuals then acquire a verbal repertoire to describe their “inner world” when in contact with verbal communities that model, shape, and reinforce descriptions related to private stimulation. For example, a young adult may start identifying as “queer” after learning the term’s meaning from others and noticing that it corresponds with their own romantic and sexual feelings. *Gender expression* can consist of overt verbal and nonverbal behavior. Conceptualized as an overt verbal response related to gender, gender expression is maintained by social contingencies when expressed to others. As an example, an individual tacting their gender expression as “transmasculine” and using “they/them” pronouns can be reinforced by a supportive verbal community. Gender expression may also serve as nonverbal behavior maintained by automatic reinforcement, given that overt and public displays of gender-nonconforming expressions can be punished in some verbal communities. For example, wearing gender-specific clothes when alone could be a nonverbal response that does not involve social mediation. In this way, gender expression is conceptually distinct from gender identity because it can be nonverbal and is typically overt verbal behavior. Finally, *sexual orientation* can be interpreted as verbal behavior about one’s own feelings, thoughts, and attraction *toward others*. Relevant controlling stimuli of verbal behavior about sexual orientation are primarily private events related to biological and physiological variables that evoke (or do not evoke) feelings of attraction and patterns of arousal in the individual.

## Potential Functions of Verbal Behavior about SOGI Identities

Verbal behavior associated with SOGI is multiply controlled, which can result in different SOGI descriptions across time, settings, and communities. An individual’s intersecting identities also contribute to the complexity of their SOGI identities and expressions. For example, a Latinx person may identify as “bisexual" among their Spanish speaking community but identify as “queer” with coworkers from the U.S. In addition, someone’s private stimulation and covert verbal behavior related to SOGI identities (e.g., feeling attraction only toward men and thinking “I’m gay”) may not correspond with their overt verbal behavior (e.g., telling others “I’m straight,”) or can vary in precision (e.g., telling others, “I like who I like.”). Variables that contribute to this lack of correspondence include different audiences (e.g., who signals a safe vs. unsafe space) and stimuli that evoke an imprecise overt verbal response (e.g., written questionnaire only gives “male” or “female” as response options so two-spirit respondent selects “female”). Thus, verbal behavior about SOGI can be influenced by positive and negative reinforcement and be maintained by social and automatic reinforcement. As an example, an individual who covertly describes themselves as “queer” may publicly identify as “queer” at an SGM-inclusive event but may hide their identity at work to avoid discrimination. This same individual receives social reinforcement from listeners at the SGM-inclusive event and automatic reinforcement from overt gender expression that aligns with their covert gender identity.

## Benefits and Risks Analysis: How to Decide whether SOGI Data Collection is Beneficial and Safe

Behavior analysts must prioritize the well-being of clients, including groups of individuals, by actively maximizing benefits and minimizing risks to them. Table 2 describes initial considerations of potential benefits and risks of SOGI data collection. Given the increased risk of harm to SGM populations, deciding whether to collect SOGI data requires a careful and thorough ethical analysis of risks and benefits to individual respondents and the larger community. The BACB requires behavior analysts to apply the Code when conducting this analysis by carefully considering the function of relevant core principles and standards, data collection context, relevant factors, and potential power imbalances of the specific situation (BACB, 2020).

**Table 2:** Initial considerations of potential benefits and risks for SOGI data collection

Benefits	*Knowledge and Needs*	Increase understanding of individuals we serve or work with Identify needs of SGM individuals
	*Culturally sensitive practices*	Provide gender-affirming care or environment Address potential inequities and barriers at the individual level
	*Procedures and policies*	Identify potential barriers to inclusivity and equity at the organizational level Inform improvement of culturally sensitive practices (e.g., trainings) in large scale
Risks	*Distress*	Elicit or evoke discomfort or emotionally challenging feelings on respondent
	*Stigmatization and discrimination*	Promote stigma and discrimination when data are handled improperly.
	*Confidentiality risk*	Raise privacy issues and compromise confidentiality when data are handled improperly.

If SOGI data collection serves no clear benefit, behavior analysts should not move forward. If SOGI data collection serves at least one clear benefit but risks of harm are identified, behavior analysts must first attempt to resolve or mitigate these risks. If all risks cannot be eliminated, behavior analysts face an ethical dilemma in which they are expected to conduct and document a structured decision-making process (BACB, 2020). Because this analysis is specific to individual contexts (National Academies of Sciences, Engineering, and Medicine, 2022), we cannot provide individualized recommendations for all situations. We recommend that behavior analysts first review the recommendations for making ethical decisions described in the Ethics Code for Behavior Analysts (BACB, 2020) and the possible risks and benefits outlined in this paper as initial considerations. Then they are tasked with using sound judgement to consider the unique aspects of their situation and recruit expertise from others as necessary.

## What are Some Benefits of Collecting SOGI Data?

### Increasing Understanding and Identifying Needs

Many behavior-analytic domains may benefit from SOGI data collection, such as clinical practice, Organizational Behavior Management (OBM), research, education, supervision, and public health. An obvious benefit is learning about respondents’ experiences and needs to better support them. Inquiring about SOGI also presents an opportunity for individuals to ask related questions or express concerns that might otherwise not be addressed. As a secondary benefit, recruiting SOGI data in an affirming-format signals acceptance and may benefit the SGM respondents’ social and emotional health (National LGBTQIA+ Health Education Center, 2022).

### Promoting Culturally Sensitive Practices

Multiple national organizations (i.e., Institute of Medicine, the Health Resources and Services Administration, the Office of the National Coordinator for Health Information Technology) recommend SOGI data collection in healthcare settings as a fundamental step toward SGM populations accessing quality healthcare (National LGBTQIA+ Health Education Center, 2022). As an example, knowing a patient is transgender allows a healthcare practitioner to provide gender-affirming care, discuss the patient’s health needs specific to their gender identity and biological anatomy, and address potential inequities or barriers at the individual level. Behavior analysts could capture similar opportunities to provide culturally competent services if aware of client’s identities.

### Improving Procedures and Policies

In public policy, SOGI data can elucidate large scale issues, inform policy decisions, and influence resource allocation (Persad, 2019). For example, accurate SOGI data can identify barriers to inclusivity, such as organizational systems reinforcing implicit bias against SGM people. It can also inform development of culturally sensitive approaches and interventions, such as the need for SGM-affirming health care coverage, pronoun-use staff training, or accessible bathrooms. Accurate SOGI data can also highlight needed resources for vulnerable groups, such as SGM groups and tutorials such as this one (Kamen et al., 2022; Streed et al., 2020).

## What are Some Risks of Collecting SOGI Data?

### Causing Distress

Behavior analysts must understand that SGM populations constitute a historically oppressed group that is particularly vulnerable to harm (APA, 2020; Capriotti & Donaldson, 2022). Not only do SGM populations have long histories of targeted violence, they continue to experience both individual and systematic discrimination today (National Academies of Sciences, Engineering, and Medicine, 2022). SGM youth experience substantially higher rates of childhood victimization, including emotional, physical, and sexual abuse, than non-SGM individuals (McGeough & Sterzing, 2018). Similarly, most SGM individuals have experienced victimization as an adult, including physical or sexual assault, theft or property destruction, and violent threats (The Williams Institute, 2019). Given these experiences, SGM individuals understandably face higher risk for mental health issues, substance abuse, suicide ideation, and completed suicide than non-SGM individuals (Coulter et al., 2019; The Trevor Project, 2020). Thus, being asked to report SOGI information may evoke negative emotional responding for SGM individuals.

### Stigmatization and Discrimination

Improper access of SOGI data could put respondents at risk of further stigmatization and discrimination. Most U.S. states do not provide legal protection from discrimination based on SGM status. Twenty-eight states do not protect SGM individuals in public accommodations, 27 states do not provide protection in housing and employment, and 32 states do not provide protection in education (The Williams Institute, 2019). If a respondent’s SGM identities were disclosed, they will likely be at higher risk of interpersonal harm, loss of public accommodations, loss of housing, loss of employment, or loss of education, without any legal recourse.

### Potential for Compromising Confidentiality

SOGI data collection and storage can raise privacy issues, especially when improper handling of data happens. Data breaches and misuse can compromise confidentiality by exposing personal information of individuals with vulnerable identities. Consequently, SGM individuals may avoid disclosing SGM identities to protect themselves from these risks. As examples, an employee may choose not to disclose their SGM identities to their employer if they work in a state that does not protect employees from employment discrimination based on SOGI. A parent may not disclose their child’s transgender identity in a state that requires mandated reporters to notify authorities when a guardian provides gender-affirming care. Similarly, a patient with a history of trauma related to their SGM identities may report imprecise SOGI data to protect themselves from further harm. The remainder of this tutorial outlines opportunities to mitigate risks and affirm SGM identities for each data practice.

## How to Collect SOGI Data

### Self-Report and Privacy

Self-report is the only socially valid method for SOGI data collection. While behavior analysts favor direct observation (Dempsey et al. 2012; Kahng et al., 2011), there are situations where direct observation is difficult, impossible, or inappropriate (Critchfield et al., 1998; Kahng et al., 2011). Biological stimulation is a primary controlling variable of individuals’ verbal behavior about SOGI and this stimulation is below the *threshold of observability* (Palmer, 2009). Similarly, while individuals of various SOGI identities may appear similar or engage in similar patterns of behavior, another person cannot identify an individual based on these observations alone. In addition, other relevant variables are context and the audience (Skinner, 1957), which can have a positive or negative reinforcement function on the behavior of reporting SOGI to others.

The behavior-analytic literature has described self-report data as potentially inaccurate or unreliable compared to “direct” measurements (see Critchfield et al., 1998, for a theoretical account on self-report). Assuming an individual’s SGM data based on their appearance or behaviors not only risks data imprecision but increases the risk of harm to the described individual. The only alternative for increasing accuracy and reliability of reported data is to design assessment procedures with stimuli that signal safety to respondents and positively reinforces disclosure of their SOGI information.

Behavior analysts should not engage in discriminatory behavior based on gender and sexual identity (BACB, Core Principle 2). Doubting someone’s identities or questioning the accuracy of someone’s SOGI self-report must not be part of the data collection process. We encourage behavior analysts to practice skepticism to improve SOGI data collection methods and to promote data quality by designing procedures that ensure respondent safety and privacy. To ethically collect SOGI data, behavior analysts must consider the surveyed population’s rights to privacy, confidentiality, and comfort when disclosing their SOGI demographics (BACB, 2020; Code 2.03, Core Principle 1). Asking an SGM individual to disclose their SOGI identities can evoke discomfort and harm given the personal nature, related histories of punishment, and potential risks from their disclosure (Maragh-Bass, 2019; National Academies of Sciences, Engineering, and Medicine, 2022).

### Risks and Protections

If the SGM individual chooses to disclose their SOGI identities, they risk real harm from bias or privacy breaches. Collecting SOGI data requires culturally competent assessors as well as robust security measures to protect against data breaches and unauthorized access. Failure to ensure that both the respondent and their data are adequately protected can result in harm and compromise trust.

Behavior analysts must ask themselves: Am I required to report SOGI data in this context? Why is it necessary, or how can it be beneficial? How will the collected information benefit the populations involved? Do my policies adequately protect respondents and their data? How will I ensure a safe space for participants to share sensitive information? How will I ethically assess, store, analyze, and report SOGI data? We strongly advise behavior analysts not to move forward with data collection until sufficient responses are provided for these questions.

### Ethics and Policies

If the behavior analyst has determined that the benefits of SOGI data collection outweigh the possible risks, they must follow the ethical standards and legal policies for their specific context. Across contexts, data procedures must protect participants from potential harm by safeguarding their SOGI data (Delpercio & Murchison, 2017). Behavior analysts must review legal and funding-source requirements that could jeopardize SOGI data protection, such as state laws that require disclosure of youths’ SGM identities to legal guardians. They must also ensure that written procedures clearly outline how SOGI data will be kept confidential, any conditions that require disclosure, and how information breaches will be addressed. Descriptions of data collection (e.g., format, procedures), storage (e.g., paper, electronically, online), and access (e.g., approved and trained personnel) can assist with evaluating preparedness (Delpercio & Murchison, 2017).

Research studies and interventions are usually tied to ethical standards, guidelines, and legal policies related to confidentiality. An important consideration is the risk SGM individuals take when disclosing their SOGI identities. Thus, we recommend behavior analysts adopt and implement clear confidentiality and nondiscrimination policies that reduce this risk for those who choose to provide this information. Assurances for children and youth are especially important because they may be at higher risk for family rejection, bullying, and sexual harassment (Delpercio & Murchison, 2017). Assurances for SGM adults are critical due to widespread prejudice and discrimination in employment and access to services (Baker et al., 2016). Please remember, over half of U.S. states do not protect SGM people from discrimination, so SGM respondents may not have legal recourse options even if they experience blatant discrimination (Movement Advancement Project, 2022).

#### Voluntary Participation and Autonomy

We must also highlight the important distinction between the need, benefit, or mandatory requirement to collect SOGI data and the respondent’s right to decline responding. Questions about SOGI identities are, by definition, personal as it relates to an individual’s sense of self. The respondents’ participation must be voluntary (National Academies of Sciences, Engineering, and Medicine, 2022). As described later in this paper, ensuring respondents autonomy requires well-informed consent and allows choices for self-identification.

## Designing SOGI Instrumentation

Well-designed SOGI data collection methods can promote a sense of representation, respect, and comfort for respondents. SOGI data can focus on an individual or a group across the lifespan and can be designed in numerous formats (Delpercio & Murchison, 2017; National Academies of Sciences, Engineering, and Medicine, 2022). These aspects must be carefully evaluated throughout SOGI data practices. Overall, the system to collect data should be (1) easy for respondents to understand and answer accurately, (2) easy for behavior analysts to understand, ask, analyze, and report data, and (3) affirm all identities by making every respondent feel included and safe (Delpercio & Murchison, 2017). Next, we describe general guidelines for behavior analysts to adapt their practices to their target population and specific context.

### Question Framing & Presentation Order

Question framing can influence whether respondents understand the question, feel safe to answer honestly, and experience feelings of affirmation or harm when participating. Given that someone’s SOGI information is only identifiable via self-report, we recommend framing questions in a way that emphasizes self-identification (e.g., “My gender identities include the following (response options listed)” or “How would you describe your gender identity?”). The goal is to frame SOGI content in an unbiased way that normalizes the fluidity and complexity of SOGI identities and allows the respondent to express their own experience with this (Suen et al., 2020).

Behavior analysts also need to consider question detail when explaining the SOGI dimension being assessed. Respondents may provide different responses about their sexual orientation if asked specifically about internal self-identification, romantic attraction, sexual attraction, or sexual behavior. They may provide different responses about their gender if asked specifically about their gender identity, gender expression, sex assigned at birth, or current anatomy (Suen et al. 2020).

For SOGI data, we generally recommend having at least two separate questions: one for sexual orientation, and another for gender identity. These are distinct aspects of someone’s identity, and each must be understood in its own context. The resulting data more precisely captures the diversity of SGM identities, validates SGM respondents, and improves representation. Medical settings and health surveys commonly use a “two-step” approach (Delpercio & Murchison, 2017) for sex and gender that includes asking about respondents’ sex assigned at birth and their current gender identities. This practice is considered by some to be more accurate for documentation purposes and can reflect nuances of transgender identities; however, questions about sex may feel invasive in non-medical settings (Delpercio & Murchison, 2017).

The presentation order of SOGI response options can contain bias. For example, it is common to see gender identity questions where “Male” is the first option. One way to make questions less biased is by presenting terms in alphabetical or randomized order.

Behavior analysts must also consider the order and placement of SOGI questions. For example, the placement of a question within the body of a survey can affect comprehension and probability of response. Using the two-step approach to collect data on sex and gender, some transgender respondents may prefer questions about their current gender identity prior to questions about their sex assigned at birth (Morgan et al., 2020) and some may prefer that SOGI questions are administered simultaneously (Suen et al., 2020). We recommend placing SOGI questions as part of the standard demographic section, which communicates that SOGI are characteristics of an individual, like race, ethnicity, and age (Badgett et al., 2014).

### Question Format & Response Options

Using open-ended questions or statements allow for the most descriptive and unbiased SOGI data collection (Delpercio & Murchison, 2017; National Academies of Sciences, Engineering, and Sciences, 2022). With this format, the survey or interviewer might ask, “What is your sexual orientation?” or “How do you describe your sexual orientation?” and allow the respondent to define their sexual orientation as they wish. A potential barrier to open-ended inquiries is that some respondents may not be familiar with relevant terminology and may provide an imprecise answer. For example, a cisgender straight individual unfamiliar with SOGI terminology could be unsure how to respond.

If the behavior analyst opts for response options, they must ensure that the question and response options match. It is common to encounter outdated or poorly informed SOGI questions that incorrectly match question and response options (National Academies of Sciences, Engineering, and Medicine, 2022). For example, a question may ask about gender but then provide only sex response options. Similarly, a question about sexual orientation may offer outdated or inaccurate response options. These discrepancies can function as microaggressions for SGM respondents. Microaggressions can be expressed in terms of subtle negative exchanges toward a marginalized individual or group (Lui & Quezada, 2019). It can have the form of verbal slights and may also be environmental in nature (Dalton & Villagran, 2018). Not having appropriate and inclusive terminology excludes identities and decreases sense of belonging.

Multiple choice is a commonly used response format. With multiple choice, the question, “Which of the following sexual orientations would you use to describe yourself? (select all that apply)” would be followed by a list of preselected response options. Anecdotal reports indicate that SOGI forms that include the respondent’s own SOGI identities can promote a sense of belonging (Delpercio & Murchison, 2017). Behavior analysts must be careful because the response option selection presents risks of bias and non-inclusivity. For example, the terms *birth sex* and *natal sex* are considered outdated (APA, 2020) while *sex assigned at birth* is a currently accepted term when referring to someone’s biological makeup. In terms of gender, offering only a few options (e.g., man, woman, transgender) or offering an “Other” option without requesting specification are considered outdated practices. These practices are non-inclusive and may cause harm to the respondent. Other outdated terms which are often considered offensive include *transgendered*, *tranny*, *transvestite*, *she-male*, *he/she*, *lady man*, *shim*, *it*, or *transsexual* (The Trevor Project, 2020). Limiting response options can contribute to misrepresentation of identities and imprecise answers. In terms of sexual orientation, it is also considered outdated to offer few options (e.g., straight, gay, or lesbian) and to use the term *homosexual* due to the historical harm associated with this term (APA, 2020, 2021). We recommend tailoring question formats and response options to the respondent population and context. For example, the age of the respondent(s) may influence their preference and choice of terms (Bauer et al., 2017). At minimum, Suen et al. (2020) recommends including asexual, pansexual, queer, and fluid for sexual orientation and gender non-conforming, nonbinary, and genderfluid for gender identity.

Behavior analysts can further improve inclusivity by including response options from diverse cultural communities. For example, Native American individuals may identify as *two-spirit* or an identity unique to their tribe (Carrier et al., 2020), and some African American SGM individuals identify as *same-gender-loving* (Brooks et al., 2022). In addition, behavior analysts might opt to tailor response options to a specific culture or audience by offering translation of terms. Part of the SOGI literature focuses on increasing construct validity of concepts and evaluating comprehension of SOGI terms across diverse cultures (Baker et al., 2016; Michaels et al., 2017; Morgan et al., 2020; Reisner et al., 2014). For example, when collecting data for community interventions, Baker and colleagues (2016) recommend translating materials into respondents’ primary languages if the population contains a significant number of non-native English speakers. Language barriers and cultural differences related to ethnic minorities can be a barrier to accurate data collection (Morgan et al., 2020). Studies with Spanish speakers and individuals from Latin American countries noted that some terms, including the distinction between sexual orientation and gender identity, did not match their own cultural conceptualization of these concepts and led to confusion (Michaels et al., 2017; Reisner et al., 2014). The remainder of this section describes further considerations for response options.

#### Write-in Answer

Considering that terms and their connotations evolve over time and across verbal communities, we suggest including a blank/self-identified option or “free-operant” response, where the respondent can write an unlisted identity or provide further information about their response (Garvey, 2019; Redford & van Wagenen, 2012). For example, if an online survey asks about gender identity, a respondent could select the “Other. Please specify:” checkbox and type in “bigender” if not included as a listed option. This additional option improves inclusivity by allowing the respondent to answer however they choose.

The write-in option also communicates an understanding that sexuality is a complex, fluid, and ever-changing concept (Garvey, 2019). It allows for the inclusion of new terms and an opportunity to analyze why respondents did not select any of the listed response options. Eliason and Streed (2017) found that some individuals may choose to write in their answer because they do not identify with or do not use SOGI labels. Notably, the wording of the write-in option is important (Redford & van Wagenen, 2012), with “Other. Please specify” preferred over “Something else”, which can be unclear or perceived as judgmental (Morgan et al., 2020).

#### Option to Not Answer

While collection of SOGI data may benefit our greater society or the respondent themselves, answers to SOGI questions must always be voluntary for the respondent (Persad, 2019). This must be clear to the individual in the initial consent and introduction of the questions (e.g., written consent forms, spoken aloud when beginning an interview). If using online surveys, we recommend deactivating forced response requirements to SOGI questions or, at minimum, adding a “Prefer not to say” option.

#### Option to Say “I don’t know”

Some individuals may not know the answer to a SOGI question and offering an “I don’t know” response option increases inclusivity. Multiple variables such as form of administration, item sensitivity, and anonymity can influence the selection of the “I don’t know” response (Morgan et al., 2020) and must, therefore, be considered when developing data collection procedures and analyzing these responses. As a potential remedy, behavior analysts may include more specific response options such as, “I don’t know what the question means,” “I am not sure about or am exploring my sexual orientation,” and “I am not comfortable answering this question.”

#### Option to Select Multiple Answers

Some individuals identify with multiple gender identities or identify with more than one term to describe their sexual orientation. For example, a person’s gender identities may include male, queer, and transgender. When developing online surveys, behavior analysts can account for this by allowing respondents to select multiple options or requesting that respondents select the option with which they identify most (The Williams Institute, 2020).

#### Design Feedback Questions

A request for feedback about the survey content or interview process may be appropriate as the last component of the assessment. Behavior analysts might include open-ended questions (e.g., “What are some comments or feedback that you have about this survey?”, “What are some ways that we can improve this interview process?”) or rating scales to evaluate the respondent’s sense of inclusion or safety (e.g., “While completing this survey about my identities, I felt included: Strongly Disagree/Disagree/don’t Disagree or Agree/Agree/Strongly Agree”). Recruiting this feedback may further communicate the behavior analyst’s desire for inclusivity, provide invaluable insight for analyses, and inform future data collection procedures.

## Administering SOGI Assessment

Once designed, the behavior analyst conducts the SOGI assessment. Ethical SOGI data collection must use self-report rather than indirect report or observation. It is critical that the assessor accept and validate the respondent’s self-reported identities. Follow-up questions may be asked for clarity, but the assessor must not question the accuracy of the reported SOGI information (e.g., if a respondent would likely be gendered as female by dominant society and states a male gender identity, the assessor documents their response and moves on). In school environments, it is important to note that state policies vary for data collection on students’ gender identities (Petrin, 2022). For example, eight states have laws that force the outing of transgender youth in schools and an additional five states have laws that promote this nonconsensual outing (Movement Advancement Project, 2024a). Six states make it a felony crime to provide gender-affirming medical care to transgender youth, and 24 states ban gender-affirming medication and surgical care (Movement Advancement Project, 2024b). In all cases and with all populations, make sure the assessor has the training and appropriate skill repertoire to ethically assess SOGI data while protecting and maintaining a safe environment for the respondent (California School Board Association, 2014; Massachusetts Department of Elementary and Secondary Education, 2021).

### Bias-Free Language

Not only must behavior analysts learn relevant SOGI terminology, they must also actively engage in bias-free language (APA, 2020). For example, if the assessment includes questions other than SOGI (e.g., title, profession, marital status, family dynamics, vignettes), the behavior analyst must ensure gender-neutral language. This is done by using inclusive nouns (e.g., *everyone*, *folks*, *partner*, *parents*, *caregivers*) instead of gendered and masculine nouns (e.g., *sir*, *ma’am*, *ladies and gentleman*, *mom and dad*). Similarly, behavior analysts must be cautious about making heteronormative assumptions (e.g., asking a female-identified person if they have a husband or assuming a child has one mother and one father).

Critically, SGM-inclusive practices should be present when interacting with respondents throughout the entire assessment process. An individual’s pronoun usage, like SOGI, cannot be assumed and must be reported by the individual about themselves. Thus, behavior analysts must either ask the individual which pronouns they use or default to gender bias-free language across both asynchronous and synchronous interactions. The APA (2020) suggests using *they*, *them*, and *theirs*, when referring to an individual who has not specified their pronouns or when gender identity is irrelevant to the context. The behavior analyst may also use only the person’s name to refer to them.

### Form of Administration and Environment

Whenever possible, we recommend that SOGI questions be self-administered. This format may increase respondents’ comfort and increase the likelihood of answering truthfully (Persad, 2019). If assessing data from groups or communities, ask questions to all individuals instead of asking based on stereotypes imposed by society about SGM individuals’ appearance and behaviors (Delpercio & Murchison, 2017). As an example, it would be inappropriate to only ask an individual about their pronouns if they disclosed a transgender identity or expressed an androgynous gender.

If self-administration is not possible, the behavior analyst must ensure that the interviewer demonstrates the appropriate cultural awareness and sensitivity to discuss SOGI with the respondent. The interview must take place in a private space where others cannot overhear the conversation or deduce the content of the interview from observing. When asking children and youth, behavior analysts should consider the influence that the presence or absence of others (e.g., caregiver, advocate, teacher) may have on the respondent’s comfort, safety, and accurate report.

### Wording Prior to Asking Questions

To assure respondents’ confidentiality and safety, we recommend full transparency when introducing SOGI questions to respondents. This includes explaining the intended purpose of data collection, who will have access, how the data will be used and kept confidential, how policies and procedures protect respondents, and how respondents can opt out of answering questions (BACB, 2020; Code 2.11). If using a survey, behavior analysts could include initial instructions similar to the following, “The next few questions ask about how you identify yourself in terms of gender and sexual orientation. This information will be used for [*purpose*]. Only [*name of person/entity*] will have access to this information. Your responses will be kept private and secure by [*method of data storage*]. At *[name of entity/organization],* we seek to provide a safe environment where differences are valued and everyone is supported across all sexual orientations and gender identities [*add other categories if applicable*]. If you don’t want to answer any of these questions, you can leave them blank or skip the question.”

If assessing SOGI information via interview, consider the level of comfort and rapport with respondents. The behavior analyst might choose to ask SOGI questions early to establish the respondent’s pronouns and inform upcoming interactions. Alternatively, the analyst might choose to ask SOGI questions at a later time to build initial rapport and trust (Delpercio & Murchison, 2017). When interviewing youth or those with cognitive deficits, behavior analysts can offer further protection by acquiring assent and providing additional detail about the policies and practices related to shared information in the specific context. For example, the behavior analyst can explain who may have access to the information (e.g., direct supervisor, some staff members who work at the clinic) and who will not have access (e.g., caregivers, teachers, friends, etc.) unless authorized by the respondent. It may be helpful to ask if there are any specific people they are concerned might learn of their responses and then directly addressing whether these people could potentially access the respondent’s data. If appropriate and the behavior analyst has systems in place, they could ask whether the respondent would like any specific person to be informed about their SOGI information, and facilitate disclosure in a safe space (e.g., a clinician assisting a client in disclosing their sexual orientation to a parent).

#### Asking SOGI Questions

To increase response accuracy in surveys, consider including a brief definition of relevant terms. As an example, a question about gender identity could be followed by the term’s definition (e.g., “Gender identity means how you describe yourself in terms of gender”). You can also include example responses (e.g., “Some examples of gender identities include: genderqueer, man, nonbinary, two-spirit, or woman.”) and include definitions of these terms as well. As an added benefit, these definitions may communicate a culture of inclusivity to all respondents and serve to educate less informed respondents of SGM identities.

With interviews, consider preparing definitions and examples similar to those described for surveys and offering this information vocally or in written form if a participant hesitates or appears confused. For example, a respondent could say, “My what?” when asked about gender identity and the interviewer could respond, “Are you familiar with the term gender identity? I can define it for you and give you some examples if that would be helpful.” These definitions, along with the initial confidentiality information and SOGI questions, can also be provided ahead of time so respondents’ can assess their comfort level and prepare their responses in a low-pressure environment. This could be especially important if using more pressured assessment methods such as live interviews or time-limited administrations.

## Analyzing SOGI Data

After collecting SOGI information, behavior analysts may then analyze and report on this data. They may encounter issues during analysis such as imprecise responses or no responses. Behavior analysts must carefully consider whether responses could have been influenced by confounding variables such as measurement error, nudge effects, or biased responding. Additionally, imprecise responses could be maintained by negative reinforcement resulting from the respondent’s history of punishment following SOGI disclosure.

### Confounding Sources of Control for Responses

If the environment feels safe to the individual and the questions are comprehensible, behavior analysts can reasonably expect SOGI data that accurately reflects respondents’ identities. However, if the individual does not feel comfortable or does not understand the questions, their responses could be controlled by confounding variables. One source of measurement error is a lack of conceptual clarity (Medina & Mahowald, 2022; National Academies of Sciences, Engineering, and Medicine, 2022). Given that SOGI terminology is constantly changing and varies across different cultures, it is possible that respondents may not understand some terms, even with definitions available.

Another source of measurement error is inadvertently influencing the respondents’ choice. An example of this error involves the order of response options influencing response selection. Thaler and Sunstein (2008) described how to deliberately “nudge” someone’s choice toward socially desirable behaviors. This strategy involved arranging the presentation of options in a way that increased the likelihood of choice toward desirable outcomes (e.g., healthy, sustainable, positive behavior). Their work highlights the impact of antecedent variables on choice and demonstrates how questionnaires can inadvertently “nudge” behavior and consequently, bias responses. Kammerer and Gerjets (2017) noted a clear bias toward users selecting the response option listed first when evaluating the effects of nudging with online users. Results from another study suggested that items viewed first were perceived of higher importance than others viewed later (Jesse & Jannach, 2021). If these findings can be generalized to SOGI research, it is possible that listing “straight” as the first response option when asking about sexual orientation could be perceived as bias and result in more respondents selecting this option. This same rationale can be applied to the first response option of any question, even when presenting items in alphabetic or randomized order, but are likely most impactful when the first option is the majority group (e.g., straight, male, cisgender). Reviewing studies on ordering effects in large scale surveys can help guide the interpretation of SOGI results in groups.

Another important source of control is the audience (Skinner, 1957) , who can evoke differentiated responding in individuals. For example, the assessor’s gender and power position can influence the respondent’s answers (Renzella et al., 2021). In face-to-face interviews, social desirability bias can influence responses to gain favor from the interviewer (Morgan et al., 2020). Self-administration and anonymous surveys are recommended to minimize these audience effects. When collecting data in communities and groups, power dynamics between community members and academic researchers can affect engagement and quality of outcomes. Efforts to improve collaborative partnership and to address inequitable dynamics may alleviate these issues (Andress et al., 2020).

To minimize measurement errors and improve data quality, we recommend pretesting SOGI data collection procedures and materials using methods such as expert reviews, focus groups, and field testing (Medina & Mahowald, 2022; Morgan et al., 2020; National Academies of Sciences, Engineering, and Medicine, 2022).

### Potential Sources of Control for Nonresponses

Nonresponses can be influenced by lack of clarity, response options, safety, or comfort when presented with SOGI questions (e.g., Brabete et al., 2020; Jans et al., 2015; Kim & Fredriksen-Goldsen, 2013). Other variables could relate to racially and ethnically driven bias (e.g., Jans et al., 2015; Kim & Fredriksen-Goldsen, 2013), and previously described variables that contribute to error in responses, including audience (e.g., Renzella et al., 2021), inequitable power dynamics (e.g., Andress et al., 2020), and measurement error. In addition, SGM individuals may not respond because they do not use labels or reject the rationale for collecting SOGI data.

Pitfalls common to surveys (e.g., break-offs, where respondents take breaks and inadvertently fail to submit all answers) can also be potential sources of control for SOGI nonresponses. Pretesting can assist with identifying and avoiding these variables prior to data collection.

## Final Remarks

Engaging in ethical SOGI data practices is one of many ways behavior analysts promote inclusion in their practices. This tutorial highlighted some considerations for behavior analysts considering data collection on sexual orientation and gender identity (SOGI), but we could not address all aspects. For example, a critical aspect of respondents that we did not thoroughly explore is the intersectionality of SGM identities with an individual’s other identities, such as race, ethnicity, culture, socioeconomic status, and disability (Crenshaw, 1989; Suen et al., 2020). These identities are not independent of one another and play into all aspects of an individual’s experience.

We strongly recommend that readers continue to seek out resources for ongoing learning due to the changing landscape and diversity of SGM experiences. In Table 3, we list some recommended resources available at the time of this paper publication. We also encourage others to build and expand this work. A future tutorial might expand on practices for maintaining cultural competence with SGM populations while a future empirical study might evaluate SGM respondents’ reported sense of inclusion following different SOGI assessment methods. We look forward to hearing more SGM voices directly sharing their experiences and knowledge of SGM inclusivity across our field and we encourage those with majority SOGI identities to elevate SGM individuals and their work whenever possible.

**Table 3:** Additional free resources on SOGI and SGM

**Description**	**Source**
Key terms about SOGI, SGM, and allyship guide	The Trevor Project (n.d.)Available at https://www.thetrevorproject.org/wp-content/uploads/2021/07/2024_Guide_Being-an-Ally-to-Trans-and-Nonbinary-Young-People.pdf
Glossary and recommendations for bias-free language	American Psychological Association (2023) Available at https://apastyle.apa.org/style-grammar-guidelines/bias-free-language/gender
Recommendations for measuring SOGI and intersectionality considerations	National Academies and of Sciences Engineering and Medicine (2022) Available at https://nap.nationalacademies.org/read/26424/chapter/1 National LGBTQIA+ Health Education Center. (2022). Available at https://www.lgbtqiahealtheducation.org/publication/ready-set-go-a-guide-for-collecting-data-on-sexual-orientation-and-gender-identity-2022-update/
	https://dpcpsi.nih.gov/sites/default/files/MethodsMeasures_Paper_508_FV.pdf

*SOGI* Sexual Orientation and Gender Identity; *SGM* Sexual and Gender Minorities. These recommendations were developed in September 2024. These recommended practices, along with the verbal behavior used to describe them, will inevitably become outdated. Readers must remain cognizant of these changes and make efforts to update their knowledge accordingly

## Data Availability

Data sharing not applicable to this article as no datasets were generated or analyzed during the current study.

## Declarations

The authors’ statements and opinions in this article are theirs and not representative of any of their affiliations.
